# In Silico Analysis: Anti-Inflammatory and α-Glucosidase Inhibitory Activity of New α-Methylene-γ-Lactams

**DOI:** 10.3390/molecules29091973

**Published:** 2024-04-25

**Authors:** Alexis Hernández-Guadarrama, Mónica Aideé Díaz-Román, Irma Linzaga-Elizalde, Blanca Eda Domínguez-Mendoza, A. Berenice Aguilar-Guadarrama

**Affiliations:** Centro de Investigaciones Químicas, IICBA, Universidad Autónoma del Estado de Morelos, Avenida Universidad 1001, Col. Chamilpa, Cuernavaca 62209, Mexico; alexis.hernandezgua@uaem.edu.mx (A.H.-G.); monica.diazr@uaem.edu.mx (M.A.D.-R.); linzaga@uaem.mx (I.L.-E.); bed@uaem.mx (B.E.D.-M.)

**Keywords:** α-methylene-γ-lactams, anti-inflammatory, α-glucosidase

## Abstract

The research about α-methylene-γ-lactams is scarce; however, their synthesis has emerged in recent years mainly because they are isosters of α-methylene-γ-lactones. This last kind of compound is structurally most common in some natural products’ nuclei, like sesquiterpene lactones that show biological activity such as anti-inflammatory, anticancer, antibacterial, etc., effects. In this work, seven α-methylene-γ-lactams were evaluated by their inflammation and α-glucosidase inhibition. Thus, compounds *3-methylene-4-phenylpyrrolidin-2-one* (**1**), *3-methylene-4-(p-tolyl)pyrrolidin-2-one* (**2**), *4-(4-chlorophenyl)-3-methylenepyrrolidin-2-one* (**3**), *4-(2-chlorophenyl)-3-methylenepyrrolidin-2-one* (**4**), *5-ethyl-3-methylene-4-phenylpyrrolidin-2-one* (**5**), *5-ethyl-3-methylene-4-(p-tolyl)pyrrolidin-2-one* (**6**) and *4-(4-chlorophenyl)-5-ethyl-3-methylenepyrrolidin-2-one* (***7***) were evaluated via in vitro α-glucosidase assay at 1 mM concentration. From this analysis, **7** exerts the best inhibitory effect on α-glucosidase compared with the vehicle, but it shows a low potency compared with the reference drug at the same dose. On the other side, inflammation edema was induced using TPA (12-*O*-tetradecanoylphorbol 13-acetate) on mouse ears; compounds **1**–**7** were tested at 10 µg/ear dose. As a result, **1**, **3**, and **5** show a better inhibition than indomethacin, at the same doses. This is a preliminary report about the biological activity of these new α-methylene-γ-lactams.

## 1. Introduction

Diabetes is a chronic medical condition characterized by elevated levels of blood glucose. This condition results from the body’s inability to effectively regulate and utilize insulin [1]. In the world, approximately 537 million adults are living with diabetes and this number is projected to rise to 643 million by 2030. Moreover, this disease and its complications cause around 1.5 million deaths worldwide [2,3]. Among the various factors contributing to the development of diabetes, some studies have demonstrated that chronic inflammation plays a key role in the pathophysiology of this disease [4,5]. Activation of several inflammatory pathways promotes the release of pro-inflammatory cytokines, which are crucial in the development of microvascular complications [6]. Currently, there is a wide variety of pharmacological options for the management of diabetes that have intrinsic anti-inflammatory effects [7]. However, due to varying responses among individuals, a significant number of these medications are associated with poor glycemic control [8]. In addition, some of them are linked to severe hypoglycemia and an increased risk of cardiovascular diseases [9,10]. These limitations underscore the need for further research and development of new bioactive molecules to improve diabetes management.

Ongoing research into the development of new antihyperglycemic agents includes lactam compounds [11,12,13]. Lactams are cyclic amides of aliphatic amino acids and form an extensive homologous series of monomers [14]. According to the number of atoms in the ring, they are classified as α-lactams, β-lactams, γ-lactams, and δ-lactams [15]. γ-lactams substituted in different positions have been used as building blocks for the synthesis of diverse bioactive compounds, and they have been investigated for their potential therapeutic applications in various diseases, including cancer, infectious diseases, and diabetes [16,17,18]. Therefore, several compounds, natural and non-natural, with a γ-lactam core in their structure have been shown to have a broad range of biological activities, including antimicrobial, antiproliferative, and anti-inflammatory activities, highlighting the potential of γ-lactams in the development of new drugs [19].

Regarding anti-inflammatory activity, some studies have revealed that the γ-lactam core could be involved in the inhibition of tumor necrosis factor-α (TNF-α), cyclooxygenase-2 (COX-2), and nuclear factor kappa B (NF-κB), which are involved in the inflammatory response [16,17,18]. Furthermore, some compounds having a γ-lactam have been shown to inhibit the formation of advanced glycation end products (AGEs) [20]. Since TNF-α, NF-κB, and AGEs are key contributors to the development and progression of diabetes, γ-lactams could be promising bioactive molecules for diabetes management. Thus, the synthesis of substituted γ-lactams and their biological evaluations are needed to determine their effectiveness as anti-inflammatory and antihyperglycemic agents. 

Herein, seven α-methylene-γ-lactams were evaluated in vivo for their anti-inflammatory activity, and in vitro for their α-glucosidase inhibitory activity. Results were correlated with in silico predictions to determine their drug-likeness properties and structure–activity relationship.

## 2. Results

### 2.1. In Silico Results

**Figure 1:**
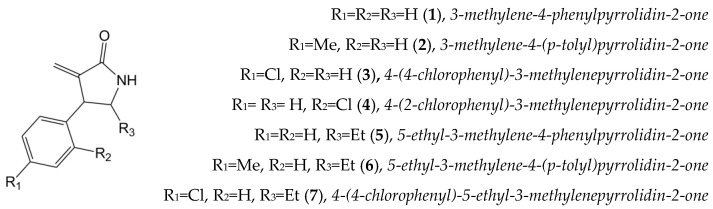
α-methylene-γ-lactams tested as α-glucosidases and inflammation inhibitors.

α-methylene-γ-lactams (**1**–**7**, Figure 1) were synthesized previously, and their data were reported by Hernández-Guadarrama et al. [21].

Previous reports have highlighted concerns about the low biological activity related to certain properties of α-methylene-γ-lactones, such as poor water solubility [22]. Therefore, in this study, the structures of the α-methylene-γ-lactams were constructed in the Molinspiration server to determine their SMILES notation (Table 1) and to predict their properties profile. This approach aimed to provide insights into their physicochemical characteristics and aid in understanding their potential therapeutic efficacy.

The properties profiles of α-methylene-γ-lactams are shown in Table 2. It is noteworthy that all seven lactams adhere to Lipinski’s rule of five, suggesting favorable absorption and permeability [23]. These observations underscore their potential as promising candidates for further exploration in drug development endeavors.

On the other hand, to predict the biological activity of α-methylene-γ-lactams, SMILES notations were introduced to the Pass Online free access program. This online server allows us to predict over 4000 types of biological activities, based on the structure–activity relationship analysis. Results are presented as a list of predicted biologic activities with the estimated probability “to be active” Pa or “to be inactive” Pi [24]. In the case of α-methylene-γ-lactams (**1**–**7**), biological activities with Pa>Pi were considered.

From this in silico prediction, it was found that **1**–**7** exhibited a Pa > Pi as anti-inflammatory and α-glucosidase inhibitors. However, all of them showed low probabilities to be active (20–31% and 13–22%, respectively). Nevertheless, it is important to note that the structure–activity relationship analysis is based on the similarity of the structure of interest to the more typical active molecules. Thus, in the case of molecules with less similarity to current anti-inflammatory or α-glucosidase inhibitors, as is the case with α-methylene-γ-lactams, there is no direct correlation between the values of Pa and the quantitative biological activity, and the chances of finding high Pa values are minimal (Table 3).

On the other hand, according to the in silico predictions, compounds **1**–**7** act as agonists of anti-inflammatory interleukins, such as IL-10, as well as antagonists of pro-inflammatory cytokines like IL-1α and IL-6 (Table 4). It is observed that all seven compounds are agonists of IL-10 with probabilities ranging from 18.9 to 27.9%. In addition, they are the antagonist of IL-1α with probabilities ranging from 8.5 to 10%. Also, lactams **4**, **5**, **6**, and **7** are antagonists of IL-6 with probabilities of 19–25.8%. Although their probability of being active as agonists or antagonists of these interleukins is low, it is important to note that their probability of being inactive is lower. Thus, taken together, these predictions allow us to suggest that the assayed α-methylene-γ-lactams could exert anti-inflammatory activity through the regulation of pro-inflammatory and anti-inflammatory cytokines.

Additionally, as is observed in Table 5, compounds **1**–**7** have an important affinity to several nicotinic acetylcholine receptors (nAChRs) subunits. On the nAChRs, the seven α-methylene-γ-lactams exhibited the highest probability of being active. The seven compounds are antagonists of the α_2_β_2_ subunit with probabilities of 65.2–87.1%. Additionally, compounds **1**, **2**, **4**, **5**, and **7** are antagonists of the α_6_β_3_β_4_α_5_ subunit, with probabilities between 66 and 78%. On the α_6_ subunit, compounds **1**, **3**, **4**, and **5** exhibited probabilities of 13.5–48.6% to be agonists, while compound **2** showed a probability of 39.8% to be an antagonist. Also, compounds **1**–**4** could act on the α_3_β_4_ subunit as agonists, with probabilities between 21.3 and 45%. On the α_4_β_4_ subunit, only compounds **1** and **3** could act as antagonists, with probabilities of 37.5% and 28.8%, respectively, while compounds **4**, **5**, and **6** could act as agonists of this subunit, with probabilities between 36 and 42.5%. Additionally, compounds **4**, **5**, and **6** are antagonists of the α_4_β_2_ subunit, with probabilities between 19 and 26.9%, and compounds **1** and **2** are agonists, with probabilities of 4.5 and 4.5%. Finally, lactams **1**, **5**, and **6** are antagonists of the α_7_ subunit, with probabilities between 6.6 and 7.6%, while lactam **6** is an agonist of this subunit with a probability of 6%.

Some of these subunits could regulate the inflammatory response through several mechanisms. Thus, given the probabilities of the biological activities of these α-methylene-γ-lactams, it is possible to suggest that they could exert anti-inflammatory activity by the regulation of interleukins through several targets, possibly the nAChRs. However, to determine these pharmacological effects, it is necessary to carry out in vivo and/or in vitro assays.

### 2.2. Anti-Inflammatory Activity 

**Figure 2:**
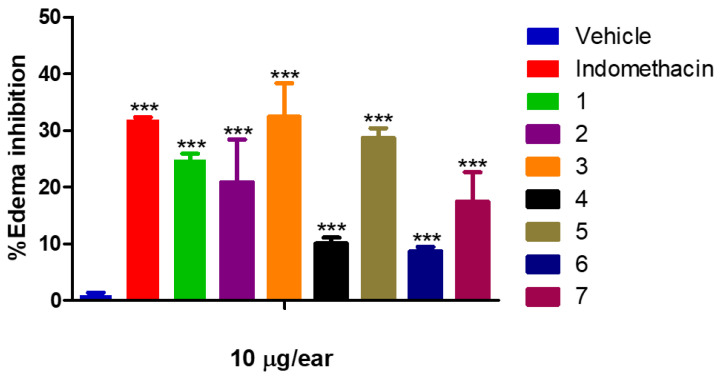
Percentage of edema inhibition of **1** (24.81 ± 1.11%), **2** (24.81 ± 7.56%), **3** (32.42 ± 5.97%), **4** (10.05 ± 1.06%), **5** (28.73 ± 1.71%), **6** (8.67 ± 0.79%), **7** (17.45 ± 5.21%), and **Indomethacin** (31.90 ± 0.06%). Dose: 10 µg/ear. Each group represents the mean ± SEM of *n* = 5, *** *p* < 0.05 vs. vehicle.

As a result of this assay (Figure 2), compound **3** (32.42 ± 5.97%) exhibited the highest efficacy in inhibiting ear edema, surpassing even the effect of indomethacin (31.90 ± 0.06%). Following closely, compounds **5** (28.73 ± 1.71%) and **1** (24.1.11%) exhibited a moderate effect on edema inhibition. Conversely, compounds **2** (24.81 ± 7.56%), **4** (10.05 ± 1.06%), **6** (8.67 ± 0.79%), and **7** (17.45 ± 5.21%) exhibited lower efficacy. These results highlight the significant impact of substituents in the γ-lactam core concerning biological activity.

### 2.3. α-Glucosidase Inhibition

**Figure 3:**
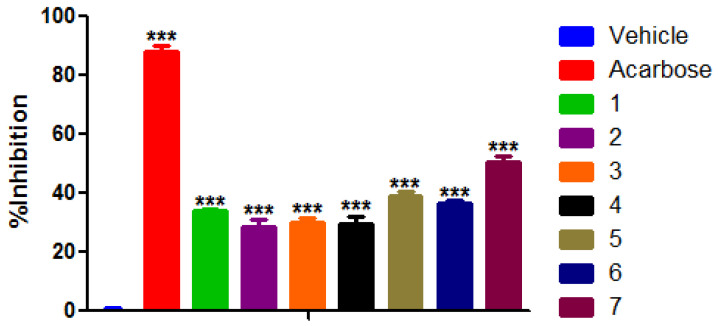
Inhibitory activity of **1** (33.91 ± 0.73%), **2** (28.80 ± 2.41%), **3** (30.31 ± 1.13%), **4** (29.37 ± 2.88%), **5** (38.88 ± 1.61%), **6** (36.36 ± 1.21%), **7** (50.68 ± 1.67%), and **Acarbose** (87.98 ± 1.86%) [1 mM] on α-glucosidase activity. Each group represents the mean ± SEM of *n* = 6, *** *p* < 0.05 vs. vehicle.

Results from the in vitro α-glucosidase inhibition assay (Figure 3) suggest that all seven lactams moderately inhibit the enzymatic activity; however, their efficacy is lower compared with acarbose (87.98 ± 1.86%). Compound **7** exhibited the highest inhibitory activity (50.68 ± 1.67%), followed by **5** (38.88 ± 1.61%), **6** (36.36 ± 1.21%), and **1** (33.91 ± 0.73%). On the other side, compounds **2** (28.80 ± 2.41%) and **4** (29.37 ± 2.88%) exhibited the lowest inhibitory effect. These findings highlight the potential of these compounds as α-glucosidase inhibitors, albeit with varying degrees of efficacy, underscoring the importance of further investigations into their therapeutic applications.

## 3. Discussion 

Diabetes represents one of the leading causes of death worldwide, with an estimated prevalence of 9.3%. Hence, it is imperative to develop novel antidiabetic agents to improve the management of this disease and mitigate its complications [25]. Given the pivotal role of inflammatory response in the development of this disease [26,27], numerous anti-inflammatory molecules have been proposed as potential antidiabetic agents [28,29,30]. Among these, γ-lactams are regarded as crucial heterocycles in medicinal chemistry due to their diverse range of biological activities. Currently, several drugs are γ-lactams derivatives, including doxapram, rolipram, and racetams [31,32]. Some studies have demonstrated their anti-inflammatory properties associated with the suppression of pro-inflammatory cytokines through different mechanisms [33,34,35,36]. α-methylene-γ-lactams are a subclass of γ-lactams that have shown potential as anti-inflammatory agents [37]. However, the development of these kinds of compounds as therapeutic agents may face challenges such as limited understanding of their biological activities, limited bioavailability, and limited in vivo and in vitro studies [38,39,40,41,42]. For this reason, further research is needed to address these challenges and determine the potential therapeutic of α-methylene-γ-lactams. In this work, seven α-methylene-γ-lactams (**1**–**7**) previously synthesized [21] were analyzed to explore their therapeutic potential.

Property profiles of these seven lactams suggest that they might have good solubility, ensuring adequate bioavailability. Furthermore, from the in silico predictions, compounds **5** and **6** showed the highest Pa as anti-inflammatory agents, while compounds **5** and **7** exhibited the highest Pa as α-glucosidase inhibitors. These three compounds have an ethyl group as a substituent in position 5 of the α-methylene-γ-lactams, while **7** has an electron-withdrawing group as an additional substituent in the para position, suggesting that these substituent groups are important for the biological activity. Thus, they were assayed in vivo and in vitro to determine their anti-inflammatory and α-glucosidase inhibitory activities.

As a result of the anti-inflammatory activity evaluation, compounds **3** and **5** exhibited the highest efficacy inhibiting ear edema, showing a behavior like indomethacin, which is an indole-based non-steroidal anti-inflammatory drug, thereby exhibiting structural similarities with the lactams assayed in this research.

Despite the structural similarities between all the assayed compounds, the position of the substituents could be key in the anti-inflammatory response. Several studies have demonstrated that the substitution with chloro (Cl), fluoro (F), methoxy (-OCH_3_), and methyl (-CH_3_) groups at the *para* position of the *N*-benzoyl group increases the anti-inflammatory activity. Additionally, the substitution in the pyrrolidine ring is important for biological activity [43]. Thus, the effect of **3** could be related to the electron-withdrawing (Cl) group in the *para* position in the aromatic ring, while the ethyl group in position 5 of compound **5** could play an important role in the interactions of this compound with targets for inflammation.

The inflammatory response induced by TPA in mouse ears involves the activation of several pathways; thus, it is difficult to predict any possible mechanisms of action for the anti-inflammatory response of these α-methylene-γ-lactams. For this reason, they were analyzed in silico. As a result, the seven lactams assayed could act as agonists of IL-10 as well as antagonists of IL-1α and IL-6 (Table 4). IL-10 regulates immune response and maintains cell homeostasis through the Jak1/Tyk2 and STAT3 signaling pathways and is considered an anti-inflammatory cytokine. Thus, the agonism of lactams on IL-10 could potentiate its anti-inflammatory activity [44]. On the contrary, IL-1α and IL-6 are pro-inflammatory cytokines; thus, their deregulation might increase the inflammatory response [45,46]. Therefore, the antagonism of the lactams on these cytokines could regulate the inflammatory response. Both effects on these cytokines could be related to the anti-inflammatory response observed in vivo.

Interestingly, these compounds exhibited a stronger affinity for some subunits of nAChRs (Table 5). These receptors have been found to play a role in the inflammatory response. Some studies have demonstrated that human T cells, monocytes, and macrophages express ACh-gated ion channels comprising several nAChRs subunits, including α_2_, α_3_, α_4_, and α_7_. For the inflammatory response, the TPA is recognized by pattern recognition receptors, like Toll-like receptors (TLRs), inducing the expression and release of several pro-inflammatory cytokines. The release of pro-inflammatory cytokines is often ATP-dependent. Thus, the activation of the ATP receptor mediates the inflammation. Several investigations have demonstrated that the activation by agonists of nAChRs subunits could inhibit the ATP receptors, downregulating the inflammatory response. However, other studies have documented that administration of phorbol ester increases the expression of some nAChRs subunits, promoting the release of pro-inflammatory cytokines and, thereby, increasing the inflammatory response [47]. Furthermore, several investigations have shown that the absence of the α_7_ subunit of nAChRs leads to metabolic disorders in mice and affects insulin release and response [48,49,50,51]. These findings suggest the agonist and antagonist effect of the nAChRs as a potential target of the α-methylene-γ-lactams assayed in this work.

However, since these receptors are implicated in several disorders, it is important to carry out more evaluations to explore new therapeutic activities. This study initiates a preliminary investigation on anti-inflammatory and α-glucosidase inhibitory activities, and the role of these receptors in inflammation pathophysiology and possibly in diabetes, one of the leading causes of death worldwide.

Since the seven α-methylene-γ-lactams showed probabilities to inhibit α-glucosidase enzymes in silico, an in vitro assay was performed. α-glucosidases are hydrolase enzymes that convert complex non-absorbable carbohydrates into simple absorbable carbohydrates, such as glucose [52]. Thus, inhibition of these enzymes is a target for diabetes management, since it delays carbohydrate absorption and reduces the rise in postprandial blood glucose concentration [53].

In the in vitro evaluation, compound **7** exhibited the most pronounced inhibitory activity, followed by compound **5**. Although their effectiveness was lower than that of acarbose, the results suggest that an ethyl substituent at the alpha position to the nitrogen as well as an electron-withdrawing group at position 4 in the aromatic ring led to enhanced inhibition compared to the electron-donating group at the same position. These findings align with the previous literature on the design of α-glucosidase inhibitors, which often involves mimicking the substrate conformation, as is the case of acarbose, and/or the positively charged transition state [54]. In this case, α-methylene-γ-lactams are positively charged due to the nitrogen atom; however, nitrogen substituents are not configurationally stable. Some studies propose that introducing a carbon chain substituent to a position adjacent to the nitrogen may stabilize the configuration and increase the α-glucosidase inhibitory activity [55].

In summary, results from this research suggest that compounds **3**, **5**, and **7** could be promising agents for the management of diabetes and inflammation. However, all compounds were assayed as a stereoisomers mixture, and their purification was not possible by conventional techniques. Thus, this is a preliminary study to determine the possible activity of α-methylene-γ-lactams, and the findings indicate that some of them are active in the targets assayed. So, the next aim will be the synthesis of these kinds of compounds as enantiomerically and diastereomerically pure and experiments modifying the substituents at the α position of nitrogen. 

## 4. Materials and Methods

### 4.1. Reagents

α-methylene-γ-lactams were previously synthesized [21]. The drugs ampicillin, acarbose, and indomethacin for the in vitro and in vivo assays were purchased from local distributors. Dimethyl sulfoxide, monosodium and disodium phosphate, as well as corn starch were products from Sigma-Aldrich (Toluca, Mexico). A glucose oxidase kit was obtained from SpinReact (Naucalpan, Mexico).

### 4.2. Animals

Male CD-1 mice (28–30 g) and male Wistar rats (200–250 g) were acquired with prior approval from the Committee for the Care and Use of Laboratory Animals (CCUAL) at the Facultad de Medicina in the Universidad Autónoma del Estado de Morelos. They were housed under standard laboratory conditions, according to the Official Mexican Rules (NOM-062-ZOO-1999) [56].

### 4.3. In Silico Predictions

Structures of a-methylene-g-lactams (**1**–**7**), previously synthesized by Hernández-Guadarrama et al. [21], are shown in Figure 1. Structures were constructed in the Molinspiration web server [57] to obtain their SMILES notation (Table 1) and properties profiles (Table 2). SMILES notation of each compound was submitted to the Pass Online [24] server to predict their biological activities.

### 4.4. In Vivo Anti-Inflammatory Assay

Anti-inflammatory activity was determined on the in vivo model of TPA-induced ear edema in mice [58]. Indomethacin was used as a reference drug. Compounds and indomethacin were assayed at 10 µg/ear dose and dissolved in an aqueous solution of DMSO (10%). Briefly, male CD-1 mice were grouped into nine groups (*n* = 5) and then anesthetized with pentobarbital sodium (0.1 UI/g). Anesthetized animals were topically administered with 10 µL of a TPA solution on both sides of the right ear. Ten minutes later, test samples were applied to the same ear. On the other side, the left ear was treated with the vehicle. Four hours after treatments, mice were sacrificed and a circumference of 6 mm diameter from both ears was obtained. The weight of auricular cuts was obtained to determine the inhibition percent of auricular edema.

### 4.5. In Vitro α-Glucosidase Inhibitory Activity

Compounds **1**–**7** were assayed in vitro to determine their α-glucosidase inhibitory activity, as previously described [59]. Briefly, Wistar rats were sacrificed by cervical dislocation to obtain the small intestine, which was flushed several times using a solution of NaCl (0.9%) and ampicillin. Consequently, a longitudinal incision was performed, and the intestinal mucosa was obtained and homogenized in an ice bath. The enzymatic activity was measured using corn starch (12.5 mg/mL) as a substrate. Acarbose was used as a reference drug. Test samples and acarbose were assayed at a concentration of 1 mM and dissolved in DMSO (30%). Initially, 125 µL of substrate, 35 µL of phosphate buffer at pH = 7, and 25 µL of test samples were added to a tube. The reactions (*n* = 6) were initiated by the addition of 50 µL of enzyme and were incubated at 37 °C for 10 min. Reactions were stopped by the addition of 2 µL acarbose and reserved in an ice bath. Quantification of free glucose was performed using a commercial glucose oxidase kit, following the manufacturer’s instructions. Finally, absorbance was measured at a 505 nm long wave.

### 4.6. Statistical Analysis

Data obtained in both experiments were analyzed in GraphPad Prism, Version 5.01. One-way ANOVA for the variance analysis followed by Dunnett’s test were performed to compare treatments vs. control. *p* < 0.05 were considered statistically significant.

## 5. Conclusions

The α-methylene-γ-lactams assessed in this research have promising physicochemical properties, as well as moderate anti-inflammatory and α-glucosidase inhibitory activities. In silico analysis provides a valuable starting point in the determination of the mechanisms of action. Thus, these structures could be useful in the development of new antidiabetic and anti-inflammatory drugs. Additionally, experimental assays on nAChRs are needed to explore different promising pharmacological activities. These multifaceted investigations serve to elucidate the comprehensive therapeutic potential of α-methylene-γ-lactams, highlighting their potential for their future applications in medical research and drug development endeavors.

## Figures and Tables

**Table 1:** SMILES notation of synthesized α-methylene-γ-lactams.

Compound	SMILES
**1**	C=C1C(=O)NCC1c2ccccc2
**2**	C=C1C(=O)NCC1c2ccc(C)cc2
**3**	C=C1C(=O)NCC1c2ccc(Cl)cc2
**4**	C=C1C(=O)NCC1c2ccccc2Cl
**5**	C=C1C(=O)NC(CC)C1c2ccccc2
**6**	C=C1C(=O)NC(CC)C1c2ccc(C)cc2
**7**	C=C1C(=O)NC(CC)C1c2ccc(Cl)cc2

**Table 2:** Properties profile of α-methylene-γ-lactams.

	α-Methylene-γ-lactams						
**Data**	**1**	**2**	**3**	**4**	**5**	**6**	**7**
**MiLogP**	1.93	2.38	2.61	2.09	2.80	3.24	3.47
**TPSA**	29.10	29.10	29.10	29.10	29.10	29.10	29.10
**Natoms**	13	14	14	14	15	16	16
**MW**	173.22	187.24	207.66	207.66	201.27	215.30	235.71
**Volume**	165.95	182.51	179.48	179.48	199.34	215.90	212.87
**nON**	2	2	2	2	2	2	2
**nOHNH**	1	1	1	1	1	1	1

MiLogP: octanol/water partition coefficient; TPSA: Topological Polar Surface Area; Natoms: number of atoms; MW: molecular weight; Volume: molecular volume; nON: H-bond acceptors; nOHNH: H-bond donors.

**Table 3:** Predicted anti-inflammatory activity and inhibition of α-glucosidase spectrum of α-methylene-γ-lactams **1**–**7** with Pass Online.

	α-Methylene-γ-lactams						
	**Anti-Inflammatory**						
**Data**	**1**	**2**	**3**	**4**	**5**	**6**	**7**
(**Pa**) (%)	25.0	-	20.0	16.0	31.0	32.0	16.0
(**Pi**) (%)	11.0	-	18.0	13.0	15.0	14.0	13.0
	**Inhibition of α-glucosidase**						
(**Pa**) (%)	6.8	16.0	11.0	13.0	21.0	13.0	17.0
(**Pi**) (%)	6.6	11.0	7.0	11.0	7.0	12.0	10.0

Pa: Probability to be active; Pi: Probability to be inactive.

**Table 4:** Predicted activity spectrum of α-methylene-γ-lactams on interleukins with Pass Online.

Receptor	Compound	Specificity	Pa (%)	Pi (%)
Interleukin 10	**1**	Agonist	27.9	2.4
	**2**	Agonist	24.4	4.6
	**3**	Agonist	22.0	6.9
	**4**	Agonist	24.7	4.3
	**5**	Agonist	24.9	4.2
	**6**	Agonist	21.0	8.0
	**7**	Agonist	18.9	10.8
Interkeukin-1α	**1**	Antagonist	8.5	5.6
	**2**	Antagonist	8.6	5.3
	**3**	Antagonist	9.9	2.8
	**4**	Antagonist	8.9	4.6
	**5**	Antagonist	8.6	5.4
	**6**	Antagonist	8.5	5.6
	**7**	Antagonist	10.0	2.6
Interleukin 6	**1**	-	-	-
	**2**	-	-	-
	**3**	-	-	-
	**4**	Antagonist	19.9	8.1
	**5**	Antagonist	24.3	4.3
	**6**	Antagonist	25.0	3.9
	**7**	Antagonist	25.8	3.4

**Table 5:** Predicted activity spectrum of α-methylene-γ-lactams on nicotinic acetylcholine receptors with Pass Online.

Receptor	Compound	Specificity	Pa	Pi
Nicotinic α_2_β_2_	**1**	Antagonist	87.1	0.4
	**2**	Antagonist	77.2	1.4
	**3**	Antagonist	78.8	1.1
	**4**	Antagonist	79.2	1.1
	**5**	Antagonist	79.5	1.1
	**6**	Antagonist	67.4	3.2
	**7**	Antagonist	65.2	3.7
Nicotinic α_6_β_3_β_4_α_5_	**1**	Antagonist	78.0	1.5
	**2**	Antagonist	66.0	4.7
	**3**	-	-	-
	**4**	Antagonist	67.3	4.3
	**5**	Antagonist	75.6	2.0
	**6**	-	-	-
	**7**	Antagonist	64.5	5.3
Nicotinic α_6_	**1**	Agonist	48.6	0.3
	**2**	Antagonist	39.8	0.5
	**3**	Agonist	37.3	0.5
	**4**	Agonist	36.9	0.5
	**5**	Agonist	13.5	7.3
	**6**	-	-	-
	**7**	-	-	-
Nicotinic α_3_β_4_	**1**	Agonist	23.8	0.7
	**2**	Agonist	21.3	1.0
	**3**	Agonist	45.0	11.1
	**4**	Agonist	22.1	0.9
	**5**	-	-	-
	**6**	-	-	-
	**7**	-	-	
Nicotinic α_4_β_4_	**1**	Agonist	37.5	17.8
	**2**	-	-	-
	**3**	Antagonist	28.8	2.6
	**4**	Agonist	42.5	13.0
	**5**	Agonist	42.3	13.2
	**6**	Agonist	36.0	19.5
	**7**	-	-	-
Nicotinic α_4_β_2_	**1**	Agonist	4.6	1.4
	**2**	Agonist	4.5	1.5
	**3**	-	-	-
	**4**	Antagonist	26.9	3.2
	**5**	Antagonist	22.1	5.4
	**6**	Antagonist	19.0	7.8
	**7**	-	-	-
Nicotinic α_7_	**1**	Antagonist	6.7	5.0
	**2**	Agonist	6.0	5.7
	**3**	-	-	-
	**4**	-	-	-
	**5**	Antagonist	7.6	4.2
	**6**	Antagonist	6.6	5.1
	**7**	-	-	-

## Data Availability

Data are contained within the article.

## References

[1] Pan American Health Organization. https://www.paho.org/en/topics/diabetes.

[2] International Diabetes Federation (2021). IDF Diabetes Atlas.

[3] World Health Organization. https://www.who.int/health-topics/diabetes#tab=tab_1.

[4] Donath M.Y., Shoelson S.E. (2011). Type 2 diabetes as an inflammatory disease. Nat. Rev. Immunol..

[5] Tsalamandris S., Antonopoulos A.S., Oikonomou E., Papamikroulis G.-A., Vogiatzi G., Papaioannou S., Defteros S., Tousoulis D. (2019). The Role of Inflammationn in Diabetes: Current Concepts and Future Perspectives. Eur. Cardiol..

[6] Ellulu M.S., Samouda H. (2022). Clinical and biological risk factors associated with inflammation in patients with type 2 diabetes mellitus. BMC Endocr. Disord..

[7] Katsiki N., Ferrannini E. (2020). Anti-inflammatory properties of antidiabetic drugs: A “promised land” in the COVID-19 era?. J. Diabetes Complicat..

[8] MacLean C.D., Littenberg B., Kennedy A.G. (2006). Limitations of diabetes pharmacotherapy: Results from the Vermont Diabetes Information System study. BMC Fam. Pract..

[9] Mattishent K., Loke Y.K. (2021). Meta-Analysis: Association between Hypoglycemia and Serious Adverse Events in Older Patients Treated with Glucose-Lowering Agents. Front. Endocrinol..

[10] Ou H.T., Chang K.C., Li C.Y. (2016). Risks of cardiovascular diseases associated with dipeptidyl peptidase-4 inhibitors and other antidiabetic drugs in patients with type 2 diabetes: A nation-wide longitudinal study. Cardiovasc. Diabetol..

[11] Goel R.K., Mahajan M.P., Kulkarni S.K. (2004). Evaluation of anti-hyperglycemic activity of some novel monocyclic beta lactams. J. Pharm. Pharm. Sci..

[12] Troisi L., Granito C., Pindinelli E. (2010). Novel and Recent Synthesis and Applications of b-Lactams. Heterocycl. Scaffolds I.

[13] Saldívar-González F.I., Lenci E., Trabocchi A., Medina-Franco J.L. (2019). Exploring the chemical space and the bioactivity profile of lactams: A chemoinformatic study. RSC Adv..

[14] Russo S., Casazza E. (2012). Ring-Opening Polymerization of Cyclic Amides (Lactams). Polym. Sci. A Compr. Ref..

[15] Das A., Banik B.K. (2020). Dipole moment in medicinal research: Green and sustainable approach. Adv. Green Sustain. Chem..

[16] Caruano J., Muccioli G.G., Robiette R. (2016). Biologically active g-lactams: Synthesis and natural sources. Org. Biomol. Chem..

[17] López-Francés A., del Corte X., Serna-Burgos Z., Martínez de Marigorta E., Palacios F., Vicario J. (2022). Exploring the Synthetic Potential of g-Lactam Derivatives Obtained from a Multicomponent Reaction—Applications as Antiproliferative Agents. Molecules.

[18] Del Corte Solaguren-Beascoa X. (2022). Multicomponent Synthesis of g-Lactam Derivatives and Applications as Anticancer Agents. Ph.D. Thesis.

[19] Harper A.D., Aitken R.A. (2020). The Chemistry of thieno [b] pyrrolones, dihydrothieno [b] pyrrolones, and their fused derivatives. Adv. Heterocycl. Chem..

[20] Jang D.S., Lee G.Y., Lee Y.M., Kim Y.S., Sun H., Kim D.H., Kim J.S. (2009). Flavan-3-ols having a gamma-lactam from the roots of Actinidia arguta inhibit the formation of advanced glycation end products in vitro. Chem. Pharm. Bull..

[21] Hernández-Guadarrama A., Cuevas F., Montoya-Balbás I.J., Román-Bravo P., Linzaga-Elizalde I. (2022). Synthesis of β-mono-and β, γ-di-substituted α-methylene-γ-lactams. Tetrahedron Lett..

[22] Woods J.R., Mo H., Bieberich A.A., Alavanja T., Colby D.A. (2013). Amino-derivatives of the sesquiterpene lactone class of natural products as prodrugs. Med. Chem. Com..

[23] Neidle S. (2012). Design Principles for Quadruplex-binding Small Molecules. Ther. Appl. Quadruplex Nucleic Acids.

[24] Filimonov D.A., Lagunin A.A., Gloriozova T.A., Rudik A.V., Druzhilovskii D.S., Pogodin P.V., Poroikov V.V. (2014). Prediction of the Biological Activity Spectra of Organic Compounds Using the Pass Online Web Resource. Chem. Heterocycl. Compd..

[25] Dhankhar S., Chauhan S., Metha D.K., Saini K., Saini M., Das R., Gupta S., Gautam V. (2023). Novel targets for potential therapeutic use in Diabetes mellitus. Diabetol. Metab. Syndr..

[26] Yousef H., Khandoker A.H., Feng S.F., Helf C., Jelinek H.F. (2023). Inflammation, oxidative stress and mitochondrial dysfunction in the progression of type II diabetes mellitus with coexisting hypertension. Front. Endocrinol..

[27] Nedosugova L.V., Markina Y.V., Bochkareva L.A., Kuzina I.A., Petunina N.A., Yudina I.Y., Kirichenko T.V. (2022). Inflammatory Mechanisms of Diabetes and Its Vascular Complications. Biomedicines.

[28] Li D., Zhong J., Zhang Q., Zhang J. (2023). Effects of anti-inflammatory therapies on glycemic control in type 2 diabetes mellitus. Front. Immunol..

[29] Pollack R.M., Donath M.Y., LeRoith D., Leibowitz G. (2016). Anti-inflammatory Agents in the Treatmet of Diabetes and Its Vascular Complications. Diabetes Care.

[30] Theofilis P., Sagris M., Oikonomou E., Antonopoulos A.S., Siasos G., Tsioufis K., Tousoulis D. (2022). The Anti-Inflammatory Effect of Novel Antidiabetic Agents. Life.

[31] Montoya-Balbas I.J., Valentin-Guevara B., López-Mendoza E., Linzaga-Elizalde I., Ordoñez M., Román-Bravo P. (2015). Efficient Synthesis of β-Aryl-γ-lactams and Their Resolution with (S)-Naproxen: Preparation of (R)-and (S)-Baclofen. Molecules.

[32] Malykh A.G., Sadaie M.R. (2010). Piracetam and piracetam-like drugs. Drugs.

[33] Kim H.K., Hwang S.-H., Oh E., Abdi S. (2017). Rolipram, a Selective Phosphodiesterase 4 Inhibitor, Ameliorates Mechanical Hyperalgesia in a Rat Model of Chemotherapy-Induced Neuropathic Pain through Inhibition of Inflammatory Cytokines in the Dorsal Root Ganglion. Front. Pharmacol..

[34] Navarro S.A., Serafim K.G.G., Mizokami S.S., Hohmann M.S.N., Casagrande R., Verri W. (2013). Analgesic activity of piracetam: Effect on cytokine production and oxidative stress. Pharmacol. Biochem. Behav..

[35] Youn D.H., Han S.W., Kim J.-T., Choi H., Lee A., Kim N., Jung H., Hong E.P., Park C.H., Lee Y. (2023). Oxiracetam alleviates anti-inflammatory activity and ameliorates cognitive impairment in the early phase of traumatic brain injury. Acta Neurochir..

[36] Zhu J., Mix E., Winblad B. (2001). The Antidepressant and Antiinflammatory Effects of Rolipram in the Central Nervous System. CNS Drug Rev..

[37] Ikuta H., Shirota H., Kobayashi S., Yamagishi Y., Yamada Y., Yamatsu I., Katayama K. (1987). Synthesis and anti-inflammatory activities of 33-(3,5-di-tert-butyl-4-hydroxybenzylidene) pyrrolidine-2-ones. J. Med. Chem..

[38] Brindisi M., Frattaruolo L., Mancuso R., Piccionello A.P., Ziccarelli I., Catto M., Nicolotti O., Altommare C.D., Gabriele B., Cappello A.R. (2021). Anticancer potential of novel α,β-unsaturated g-lactam derivatives targeting the PI3K/AKT signaling pathway. Biochem. Pharmacol..

[39] Liu S., Zhuang Z., Qiao J.X., Yeung K.-S., Su S., Cherney E.C., Ruan Z., Ewing W.R., Poss M.A., Yu J.-Q. (2021). Ligand Enabled Pd(II)-Catalyzed g-C(sp^3^)-H Lactamization of Native Amides. J. Am. Chem. Soc..

[40] Erbay T.G., Dempe D.P., Godugu B., Liu P., Brummond K.M. (2021). Thiol Reactivity of N-Aryl-a-methylene-g-lactams: A Reactive Group for Targeted Covalent Inhibitor Design. J. Org. Chem..

[41] Pieber B., Gilmore K., Seeberger P.H. (2017). Integrated Flow Processing—Challenges in Continuous Multistep Synthesis. J. Flow Chem..

[42] del Corte X., López-Francés A., Maestro A., Villate-Beitia I., Sainz-Ramos M., Martínez de Marigorta E., Pedraz J.L., Palacios F., Vicario J. (2021). A Multicomponent Protocol for the Synthesis of Highly Functionalized g-Lactam Derivatives and Their Applications as Antiproliferative Agents. Pharmaceuticals.

[43] Bian M., Ma Q.-Q., Wu Y., Du H.-H., Guo-Hua G. (2021). Small molecule compounds with good anti-inflammatory activity reported in the literature from 01/2009 to 05/2021: A review. J. Enzym. Inhib. Med. Chem..

[44] Carrasco-Serrano C., Viniegra S., Ballesta J.J., Criado M. (2000). Phorbol ester activation of the neuronal nicotinic acetylcholine receptor alpha7 subunit gene: Involvement of transcription factor Egr-1. J. Neurochem..

[45] Carlini V., Noonan D.M., Abdalalem E., Goletti D., Sansone C., Calabrone L., Albini A. (2023). The multifaceted nature of IL-10: Regulation, role in immunological homeostasis and its relevance to cancer, COVID-19 and post-COVID conditions. Front. Immunol..

[46] Di Paolo N.C., Shayakhmetov D.M. (2016). Interleukin 1α and the inflammatory process. Nat. Immunol..

[47] Luo Y., Zheng S.G. (2016). Hall of Fame among Pro-inflammatory Cytokines: Interleukin-6 Gene and Its Transcriptional Regulation Mechanisms. Front. Immunol..

[48] Li J., Mathieu S.L., Harris R., Ji J., Anderson D.J., Malysz J., Bunnelle W.H., Waring J.F., Marsh K.C.Ç., Murtaza A. (2011). Role of a7 nicotinic acetylcholine receptors in regulating tumor necrosis factor-α (TNF-α) as revealed by subtype selective agonist. J. Neuroimmunol..

[49] Fernández-Cabezudo M.J., George J.A., Bashir G., Mohamed Y.A., AI-Mansori A., Qureshi M.M., Lorke D.E., Petroianu G., Ramadi B.K. (2019). Involvement of Aceylcholine Receptors in Cholinergic Pathway-Mediated Protection Against Autoimmune Diabetes. Front. Immunol..

[50] Ganic E., Singh T., Luan C., Fadista J., Johansson J.K., Cyphert H.A., Bennet H., Storm P., Prost G., Ahlenius H. (2016). MafA-Controlled Nicotinic Receptor Expression Is Essential for Insulin Secretion and Is Impaired in Patients with Type 2 Diabetes. Cell Rep..

[51] Gausserès B., Liu J., Foppen E., Tourrel-Cuzin C., Rodriguez Sanchez-Archidona A., Delangre E., Cruciani-Guglielmacci C., Pons S., Maskos U., Thorens B. (2020). The Constitutive Lack of α7 Nicotinic Receptor Leads to Metabolic Disorders in Mouse. Biomolecules.

[52] Tomasik P., Horton D. (2012). Chapter 2—Enzymatic Conversions of Starch. Advances in Carbohydrate Chemistry and Biochemistry.

[53] Khoo C.M. (2017). Diabetes Mellitus Treatment. International Encyclopedia of Public Health.

[54] Tseng P., Ande C., Moremen K.W., Crich D. (2023). Influence of Side Chain Conformation on the Activity of Glycosidase Inhibitors. Angew. Chem. Int. Ed..

[55] Trapero A., Llebaria A. (2012). A Prospect for Pyrrolidine Iminosugars as Antidiabetic α-Glucosidase Inhibitors. J. Med. Chem..

[56] Norma Oficial Mexicana NOM-062-ZOO-1999 Diario Oficial de la Federación, México. 15. Organización Munndial de Sanidad Animal.

[57] Molinspiration Cheminformatics Free Web Services, Slovensky Grob, Slovakia. https://www.molinspiration.com.

[58] García-Argáez A.N., Ramírez-Apan T.O., Parra-Delgado H., Velázquez G., Martínez-Vázquez M. (2000). Anti-inflammatory Activity of Coumarins from Decatropis bicolor on TPA Ear Mice Model. Plant Med..

[59] Ramírez G., Zavala M., Pérez J., Zamilpa A. (2012). In vitro screening of medicinal plants used in Mexico as antidiabetics with glucosidase and lipase inhibitory activities. Evid. Based Complement. Alternat. Med..

